# Acute Myeloid Leukemia in an Infant with t(8;19)(p11.2;q13) Translocation: Case Report and a Review of the Literature

**DOI:** 10.1155/2019/4198415

**Published:** 2019-09-08

**Authors:** Ashley C. Eason, Silvia T. Bunting, Jess F. Peterson, Debra Saxe, Himalee S. Sabnis

**Affiliations:** ^1^Department of Pediatrics, Aflac Cancer and Blood Disorders Center, Children's Healthcare of Atlanta and Emory University, Atlanta, GA, USA; ^2^Department of Pathology, Children's Healthcare of Atlanta, Atlanta, GA, USA; ^3^Department of Laboratory Medicine and Pathology, Mayo Clinic, Rochester, MN, USA; ^4^Department of Pathology and Laboratory Medicine, Emory University, Atlanta, GA, USA

## Abstract

Acute myeloid leukemia (AML) patients with t(8;16)(p11.2;p13) constitute a small subgroup with a distinct genetic and clinical profile. We present a unique case of a female infant with monocytic AML associated with t(8;19)(p11.2;q13.3), a rarely reported variation of t(8;16)(p11.2;p13). The patient presented with leukemia cutis and demonstrated erythrophagocytosis in the diagnostic bone marrow. She responded well to standard AML chemotherapy and is currently in remission. Here, we highlight her case as the youngest AML patient with t(8;19) described in the literature, discuss the significance and prognostic implications of this genetic variant, and review 8p11.2 fusion proteins in AML.

## 1. Introduction

In acute myeloid leukemia (AML), genetic mutations are often associated with specific disease subtypes which have biologic and prognostic implications [1]. While AML continues to be a predominantly adult disease, it is the second most common acute leukemia in children and is associated with high morbidity and mortality. Among the cytogenetic abnormalities seen in AML, translocation between 8p11.2 and 16p13 is a rare gene rearrangement seen in <1% of adult and pediatric patients [2–4]. It results in fusion of *KAT6A* (lysine acetyltransferase 6A) with *CREBBP* (CREB-binding protein), and the gene expression profile of these leukemias resembles that of AML with MLL (mixed lineage leukemia) gene rearrangement [5]. Additionally, it is characterized by a monocytic phenotype, leukemia cutis at presentation, histologic evidence of erythro/hemophagocytosis and spontaneous remission, specifically in congenital cases [3, 4, 6, 7]. Aside from transient myelopoiesis associated with Down's syndrome, this is the only described self-limited neonatal leukemia [3]. However, in adults, t(8;16)(p11.2;p13) AML has been shown to have a poor prognosis, which may be related to the increased frequency of this translocation with therapy-related AML [4].

AML associated with t(8;19)(p11.2;q13) is an extremely rare variant of 8p11.2-rearranged acute leukemias. Although infrequently reported in the literature, it is believed to behave similarly to t(8;16)(p11.2;p13) AML [7–10]. We report a case of a 3-month-old female infant presenting with skin nodules, who upon further evaluation was confirmed to have leukemia cutis and a diagnosis of AML with myelomonocytic differentiation. Cytogenetic evaluation demonstrated t(8;19)(p11.2;q13). This rare translocation has been previously described in only four patients in the literature. Here, we highlight the youngest reported case of t(8;19)(p11.2;q13) AML and review the disease characteristics and clinical outcomes associated with 8p11.2-rearranged leukemias.

## 2. Case Description

**Figure 1:**
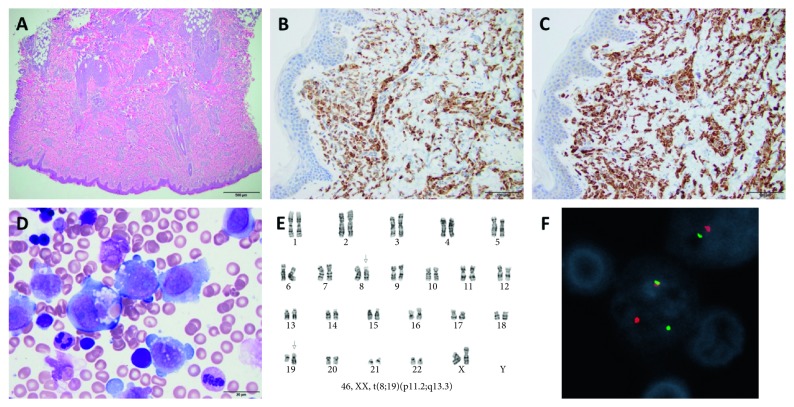
Histopathology and cytogenetic features of the case. (a) Skin biopsy shows diffuse infiltrates in the dermis and subcutis, concentrated around the vessels and appendages. (b and c) Cells are positive for CD68 and myeloperoxidase, respectively. (d) Blasts show evidence of erythrophagocytosis. (e) Patient karyotype demonstrating t(8;19) (p11.2;q13.3). (f) Representative interphase cell demonstrating a *KAT6A* rearrangement by fluorescence *in situ* hybridization.

A 3-month-old Caucasian female presented to her pediatrician with a spreading erythematous maculopapular rash with bluish raised lesions, most prominent over her back and scalp. Her first skin lesion was noted shortly after she received vaccinations at 2 months of age. A skin biopsy was notable for a monocytic infiltrative process consistent with leukemia cutis (Figures 1(a)–1(c)). She was subsequently referred to pediatric oncology for further evaluation and treatment. Her initial complete blood count showed a normal leukocyte count (14.63 × 10^3^/*μ*L) with a normal differential, hemoglobin (11.1 g/dL) and platelet count (281 × 10^3^/*μ*L). Flow cytometry of the bone marrow aspirate revealed a distinct population of cells totaling 26% of the sample, expressing bright CD4, CD11c, dim CD13, CD15, CD16, and CD33, CD36, CD38, CD58, CD123, HLA-DR, myeloperoxidase (MPO), dim terminal deoxynucleotidyl transferase (TdT), and moderate CD45. These findings confirmed the diagnosis of AML with monocytic differentiation (French-American-British (FAB) M4). Striking erythrophagocytosis was noted in the bone marrow biopsy (Figure 1(d)). Chromosome analysis showed translocation between the short arm of chromosome 8 and the long arm of chromosome 19 with resulting karyotype t(8;19)(p11.2;q13) (Figure 1(e)). Fluorescence *in situ* hybridization (FISH) was performed on a bone marrow aspirate smear using a *KAT6A* break-apart probe set (CytoTest Inc., Rockville, MD), confirming a *KAT6A* rearrangement in metaphases (seen in 50% of 200 interphase nuclei analyzed) (Figure 1(f)).

The patient was treated per our institutional standard of care for AML patients. She received induction therapy with ADE which included cytarabine 3.3 mg/kg every 12 hours for days 1–10, daunorubicin 1.7 mg/kg for three doses on days 1, 3, and 5, and etoposide 3.3 mg/kg daily for days 1–5. She had no evidence of minimal residual disease (MRD), defined as <0.1% disease in bone marrow by flow cytometry, at the end of induction. Her subsequent chemotherapy included three more cycles of chemotherapy: Induction II with cytarabine 33 mg/kg every 12 hours on days 1–4 and mitoxantrone 0.4 mg/kg daily on days 3–6, Intensification I with cytarabine 33 mg/kg every 12 hours on days 1–5 and etoposide 85 mg/kg daily on days 1–5, and Intensification II with cytarabine 100 mg/kg every 12 hours on days 1-2 and 8-9 and Erwinia asparaginase 830 units/kg on days 2 and 9. Per institutional standard, she remained hospitalized following chemotherapy through count recovery. She tolerated each course of chemotherapy well with minimal toxicity and continues to remain in remission now two years from initial diagnosis.

## 3. Discussion

Cytogenetic abnormalities play a prominent role in risk stratification of AML, with t(8;16)(p11.2;p13)-associated AML demonstrating a unique phenotype [4]. This translocation creates a fusion of *KAT6A* (previously *MOZ* or *MYST3*) gene on chromosome 8 with the *CREBBP* gene on chromosome 16 [4]. Both *KAT6A* and *CREBBP* modulate gene transcription and induce hematopoietic cell transformation [11]. In adults, this rare translocation has been noted with increased frequency in patients with therapy-related AML compared to patients with *de novo* AML [4, 12]. Most patients with t(8;16) AML demonstrated M4/M5 FAB subtype, erythrophagocytosis, and a gene signature similar to *MLL* (*KMT2A*)-rearranged leukemias. Median overall survival in two large cohorts was poor (4.7–8.5 months), which may be related to the high number of therapy-related AML cases most often seen following treatment for solid tumors in these groups [4, 12]. In addition, the majority of patients in these cohorts were adults with only two pediatric cases reported [4, 12]. Interestingly, both were infants less than 6 months of age [12].

Similar to adults, pediatric AML with t(8;16)(p11.2;p13) has been characterized as a distinct subgroup commonly associated with leukemia cutis and hemophagocytosis at diagnosis. In the largest pediatric evaluation of this translocation to date, the International Berlin-Frankfurt-Munster (I-BFM) AML study group reviewed 62 patients with t(8;16)(p11.2;p13) and compared them with a pediatric AML reference cohort [3]. Nearly all patients had *de novo* disease (60/62) and presented with M4/M5 FAB AML (97% compared to only 41% in the reference group). Leukemia cutis occurred more commonly in infants and with higher frequency (58% versus 8%) as compared to other AML patients. Selective activation of HOXA (homeobox A cluster) genes without activation of HOXB (homeobox B cluster) genes was noted in gene expression profiling, similar to *KMT2A*-rearranged AML. Disseminated intravascular coagulation (DIC) at presentation was noted in 15/38 of the t(8;16)(p11.2;p13) patients at diagnosis. A substantial subgroup of patients (17/62) was diagnosed in the first month of life which was significantly higher than the reference cohort. Seven of these seventeen infants were initially untreated and noted to have a spontaneous remission. Four patients subsequently developed recurrence requiring treatment; however, 5/7 patients were alive at last follow-up. Five-year overall survival rate for the entire cohort was 59% (±9%) similar to the rate for the reference group (62% ± 2%).

While t(8;16)(p11.2;p13)-associated AML is described in several studies, AML involving t(8;19)(p11.2;q13) has been rarely reported and is considered a subgroup of the t(8;16) subtype. In 1988, Brizard et al. first noted the t(8;19)(p11.2;q13) variant in an 8-month-old infant with M5 AML and marked erythrophagocytosis [8]. The infant achieved remission following standard chemotherapy, but later died due to veno-occlusive disease following hematopoietic stem cell transplantation. Since then, three other patients have been reported (Table 1) [7, 9, 10]. To the best of our knowledge, this is the youngest patient to be described in the literature harboring this translocation. Most of these patients (3/5) were males and had *de novo* AML (4/5). Other coexisting cytogenetic mutations were rare (1/5), suggesting this is likely an initiating event in leukemogenesis. All patients with *de novo* disease had a favorable response to standard chemotherapy alone, and only one patient underwent stem cell transplantation. The one patient with therapy-related AML did not respond to initial chemotherapy and died shortly after.

The fusion partner of the *KAT6A* gene in t(8;19)(p11.2;q13) was recently identified in a patient with therapy-related AML to be the leucine twenty homeobox (*LEUTX*) gene located on chromosome 19q13 [10]. It plays an important role in preimplantation embryo development and is known to be primarily expressed in human embryos; however, the exact mechanism of leukemogenesis in t(8;19)(p11.2;q13) AML remains unclear [13]. In addition to *CREBBP* and *LEUTX*, four other *KAT6A* fusion partners have been identified, namely, *EP300*, *NCOA2*, *NCOA3*, and *ASXL2* [14]. Similar to *CREBBP*, all except *ASXL2* and *LEUTX* contain a histone acetyltransferase domain. Cases involving *KAT6A-CREBBP* fusions have demonstrated gain of *MYB* and overexpression of *HOXA9*, *HOXA10*, *HOXA11*, *CEBPA*, *LMO2*, and *PTPN6* genes [14]. *C-MYB* gene is a downstream target of *HOXA9* and upregulation of *HOXA9*, and other *HOX* genes have been implicated in leukemic transformation via increased self-renewal of leukemic stem cells [15]. *KAT6A* leukemias are classified with M4/M5 AML with the exception of those having *ASXL2* as a partner [10].

AML associated with t(8;19)(p11.2;q13) remains a rarely described entity in both pediatric and adult patients. Similar to other infant cases of AML with t(8;16)(p11.2;p13), our patient presented with leukemia cutis, demonstrated erythrophagocytosis in the bone marrow, and was able to achieve remission with standard AML therapy supporting the biologic similarities between the two disease subtypes. While there likely are similar underlying pathways for leukemogenesis between the two 8p11.2-translocated subgroups, our case suggests that the t(8;19) variant is associated with a favorable prognosis in pediatric patients, similar to previously reported cases of t(8;19)(p11.2;q13) AML.

## Figures and Tables

**Table 1:** Summary of t(8;19)(p11.2;q13) cases in the literature.

Age/sex	Disease	FAB	Karyotype	AML treatment	Outcome	Reference (year)
8 months/M	*De novo*	M5	46,XY,t(8;19)(p11.2;q13.2) [8]/46,XY,t(8;19), −1,+1q+[22]/46,XY,t(8;19),−16,+16q+[6]	Chemotherapy ⟶ BMT	Remission ⟶ death due to VOD	Brizard et al. [8] (1988)
15 years/F	*De novo*	M4	46,XX,t(8;19)(p11;q13) [6]/46,XX [15]	Chemotherapy alone	Remission (14 months)	Stark et al. [9] (1995)
76 years/M	*De novo*	M5a	46,XY,t(8;19)(p11;q13.3)[18]/46,XY [2]	Chemotherapy alone	Remission (8 months)	Gervais et al. [7] (2008)
71 years/M	Secondary AML	M4	46,XY,t(8;19)(p11;q13)[20]	Chemotherapy alone	No response to therapy—death due to disease progression	Chinen et al. [10] (2014)
3 months/F	*De novo*	M4	46,XX,t(8;19)(p11.2;q13.3)[16]/46,XX [4]	Chemotherapy alone	Remission (24 months)	Current case

FAB, French-American-British classification; AML, acute myeloid leukemia; BMT, bone marrow transplantation; VOD, veno-occlusive disease.
